# NDUFAF5 Hydroxylates NDUFS7 at an Early Stage in the Assembly of Human Complex I*[Fn FN2]

**DOI:** 10.1074/jbc.M116.734970

**Published:** 2016-05-18

**Authors:** Virginie F. Rhein, Joe Carroll, Shujing Ding, Ian M. Fearnley, John E. Walker

**Affiliations:** From the Medical Research Council Mitochondrial Biology Unit, Cambridge Biomedical Campus, Cambridge CB2 0XY, United Kingdom

**Keywords:** bioenergetics, Complex I, electron transport, hydroxylase, mitochondria, assembly, methyltransferase, protein hydroxylation

## Abstract

Complex I (NADH ubiquinone oxidoreductase) in mammalian mitochondria is an L-shaped assembly of 45 proteins. One arm lies in the inner membrane, and the other extends about 100 Å into the matrix of the organelle. The extrinsic arm contains binding sites for NADH, the primary electron acceptor FMN, and seven iron-sulfur clusters that form a pathway for electrons linking FMN to the terminal electron acceptor, ubiquinone, which is bound in a tunnel in the region of the junction between the arms. The membrane arm contains four antiporter-like domains, energetically coupled to the quinone site and involved in pumping protons from the matrix into the intermembrane space contributing to the proton motive force. Seven of the subunits, forming the core of the membrane arm, are translated from mitochondrial genes, and the remaining subunits, the products of nuclear genes, are imported from the cytosol. Their assembly is coordinated by at least thirteen extrinsic assembly factor proteins that are not part of the fully assembled complex. They assist in insertion of co-factors and in building up the complex from smaller sub-assemblies. One such factor, NDUFAF5, belongs to the family of seven-β-strand *S*-adenosylmethionine-dependent methyltransferases. However, similar to another family member, RdmB, it catalyzes the introduction of a hydroxyl group, in the case of NDUFAF5, into Arg-73 in the NDUFS7 subunit of human complex I. This modification occurs early in the pathway of assembly of complex I, before the formation of the juncture between peripheral and membrane arms.

## Introduction

Mammalian complex I (NADH:ubiquinone oxidoreductase)^2^ provides the entry point for electrons from NADH into the mitochondrial electron transport chain, and couples electron transfer through the complex to the generation of the proton-motive force across the inner membrane (1, 2). It is composed of forty-five proteins with a combined mass of about 1 MDa, assembled into an L-shaped complex, with one arm embedded in the inner membranes of mitochondria and the orthogonal, or peripheral, arm extending into the matrix of the organelle (3–5). Seven of the subunits, named ND1-ND6 and ND4L, are hydrophobic proteins that emanate from the mitochondrial genome (6, 7), and the remainder are encoded in the nucleus and are imported into the organelle (8). The seven mitochondrial-encoded subunits and seven of the nuclear-encoded subunits form the catalytic core of the enzyme (5, 9). Electrons are transferred one at a time from NADH through the peripheral arm via the primary electron acceptor, FMN, and a chain of seven iron-sulfur clusters, to the terminal acceptor, ubiquinone. The oxidized ubiquinone binds in the region of the juncture between the peripheral and membrane arms (9, 10), and transfer of two electrons, one at a time, from the terminal electron acceptor iron-sulfur cluster N2 to ubiquinone is coupled to the ejection of four protons from the mitochondrial matrix into the inter-membrane space, thereby contributing to the generation of the proton motive force (11, 12). Proton translocation takes place in the membrane arm, probably via four antiporter folds. The remaining thirty supernumerary subunits are distributed between both arms. They have no direct roles in catalysis, but at least one of them is involved in the assembly of the complex (13). A structure of bovine complex I at 5 Å resolution contains the fourteen core subunits modeled on the basis of a high resolution structure of the bacterial complex, and in addition the folds of twenty-two supernumerary subunits have been recognized (5, 14). The structure of complex I from *Yarrowia lipolytica* has been resolved in a similar way (15).

The pathway of biogenesis of human complex I proceeds via the formation of specific intermediate sub-complexes (16, 17), and at least thirteen protein assembly factors, that are not constituents of the mature functional enzyme, participate in this process (13, 18). One of them, NDUFAF7, is a member of the 7β-strand family of *S*-adenosylmethionine (SAM)-dependent methyltransferases, and transfers methyl groups to the ω-N^G^- and ω-N^G′^-atoms of Arg-85 in subunit NDUFS2 at an early stage in the assembly of complex I (19, 20). NDUFAF5 is another member of the 7β-strand family of SAM-dependent methyltransferases. Its biochemical function is not known, but pathogenic mutations in NDUFAF5 are associated with a deficiency in complex I, and are found in patients with neonatal mitochondrial disease (21) and Leigh syndrome (22, 23). The introduction of these mutations into *Dictyostelium* produced a defect in complex I similar to that observed in human cells (24). Here, we describe investigations into the role of NDUFAF5 in human mitochondria.

## Results

### 

#### 

##### Sub-cellular Location of NDUFAF5

**FIGURE 1.**
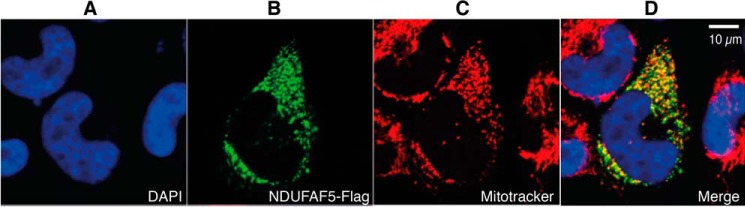
**Sub-cellular location of NDUFAF5.** Human 143B cells were transfected with a plasmid encoding tagged NDUFAF5, and immunocytometry was performed 24 h later. *Part A*, cell nucleus stained with DAPI (*blue*); *part B*, recombinant NDUFAF5 detected with an anti-FLAG antibody, plus goat anti-mouse Alexa Fluor 488 (*green*); *part C*, mitochondria stained with MitoTracker (*red*); *part D*, merged areas of *parts A–C*.

NDUFAF5 with a C-terminal FLAG tag was found uniquely in the mitochondria of human 143B cells (Fig. 1), in agreement with earlier experiments (21).

##### Interaction of NDUFAF5 with Subunit NDUFS7 of Complex I

**FIGURE 2.**
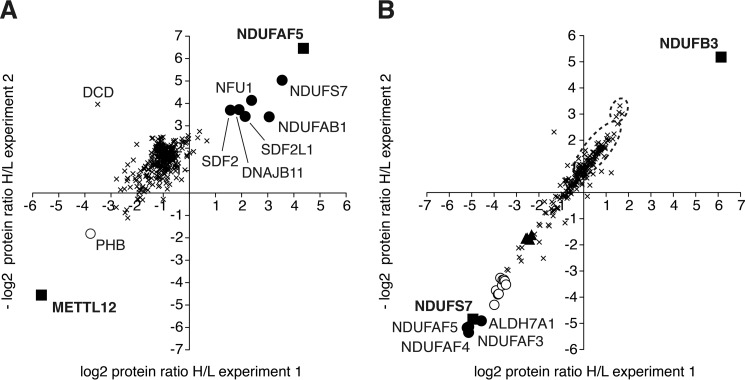
**Proteins associated with NDUFAF5 and NDUFS7.** *Parts A* and *B*, quantitative mass spectrometry of NDUFAF5 and NDUFS7 and associated proteins, respectively. SILAC-labeled HEK293T cells overexpressing tagged and control proteins were grown, and then tagged and associated proteins were purified and combined. *A*, tagged-NDUFAF5 and differentially labeled control cells overexpressing tagged-METTL12, and, in *B*, tagged-NDUFS7 and differentially labeled control cells overexpressing tagged-NDUFB3. The experiments were performed in both labeling orientations. *Part A*, ■, NDUFAF5 and METTL12; ●, proteins significantly associated in both experiments with NDUFAF5, and ○, with METTL12; ×, 312 insignificantly associated proteins, and 8 associated significantly in one orientation of labeling only; DCD, exogenous contaminant (dermcidin). *Part B*, ■, NDUFS7, and NDUFB3; ●, proteins significantly associated with NDUFS7 in both experiments; ×, 203 insignificantly associated proteins, and ○, 10 proteins significantly associated with overexpressed NDUFS7 in one labeled-orientation only; ▴, complex I subunits NDUFS2, NDUFS3, NDUFS8, and NDUFA5; the larger *black dotted ellipse* encompasses 26 other subunits of complex I, and the smaller ellipse contains four subunits of complex III (see supplemental Table S2).

By SILAC and quantitative mass spectrometry experiments with an inducible tagged version of NDUFAF5, six proteins were found to be associated with statistical significance with NDUFAF5 (Fig. 2*A* and supplemental Table S1). The complex I subunit, NDUFS7, was enriched to the greatest extent, and the complex I subunit NDUFAB1, iron-sulfur cluster scaffold homolog NFU1, stroma cell-derived factors 2 and 2-like (SDF2 and SDF2L1, respectively), and DNAJ homolog subfamily B member 11 (DNAJB11), to lesser degrees. Control experiments were conducted in parallel with an inducible and tagged version of METTL12, a member of the 7β-strand methyltransferases of unknown function. Complementary SILAC experiments were performed with a tagged version of NDUFS7 and with a tagged version of the complex I subunit NDUFB3 as a control. They showed that NDUFAF5, NDUFAF3, and NDUFAF4 (both assembly factors for complex I) and α-aminoadipic semialdehyde dehydrogenase (ALDH7A1) were associated significantly with NDUFS7 (Fig. 2*B* and supplemental Table S2), and that no protein was associated significantly with NDUFB3. However, in both of these independent and complementary experiments, NDUFS7 and NDUFAF5 were the only two proteins that were associated consistently with each other.

##### Suppression of NDUFAF5 and the Hydroxylation of Arg-73 in NDUFS7

**FIGURE 3.**
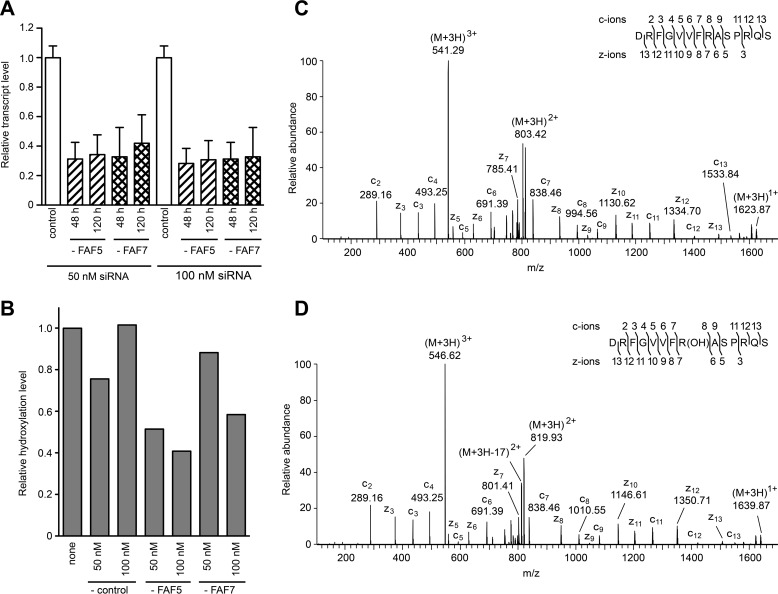
**Effect of suppression of expression of NDUFAF5 on hydroxylation of Arg-73 in NDUFS7.** *Part A*, HEK293T cells overexpressing tagged-NDUFS7 were transfected twice at 0 and 72 h, with 50 or 100 nm siRNA specific for NDUFAF5 (-FAF5), NDUFAF7 (-FAF7), or with a negative control siRNA. Transcript levels were examined at 48 and 120 h and are normalized to endogenous β-actin. The error bars show the standard deviation. *Part B*, mass spectrometric analysis of the hydroxylation of Arg-73 in NDUFS7. The histograms are derived from the extracted ion chromatograms for the *m*/*z* values of the hydroxylated or non-hydroxylated Asp-N peptide (residues 66–79) from NDUFS7. The numbers correspond to the level of hydroxylation relative to a value of 1.0 for the untreated control. *Parts C* and *D*, fragmentation spectra of triply charged ions, *m*/*z* 541.29 and *m*/*z* 546.62, representing unmodified and hydroxylated versions of the AspN peptide (resides 66–79) from human NDUFS7. In the *insets*, fragment ions are mapped onto the amino acid sequence.

Subunit NDUFS7 contains no post-translationally methylated amino acids, but the conserved arginine residue (residues 73 and 77 in the human and bovine complexes, respectively) is completely hydroxylated (25). Therefore, the possibility that NDUFAF5 might catalyze the introduction of this modification was investigated as follows. In HEK cells where tagged-NDUFS7 was being overexpressed, the expression of NDUFAF5 was suppressed transiently, and in a control experiment the expression of the assembly factor NDUFAF7 was suppressed. It was found that the transcripts for NDUFAF5 and NDUFAF7 had been suppressed by 60–70% relative to controls, and these levels of suppression had been maintained throughout the experiment (Fig. 3*A*). In untreated cells where NDUFS7 had been overexpressed, about 50% of its Arg-73 was hydroxylated, and treatment with negative control siRNA did not alter this level appreciably (Fig. 3*B*). However, suppression of the expression of NDUFAF5 with 50 nm siRNA reduced the relative hydroxylation of Arg-73 by 49%, whereas suppression of NDUFAF7 had no impact on hydroxylation of this residue (Fig. 3*B*). When the expression of NDUFAF5 was suppressed with a higher 100 nm concentration of siRNA the relative level of hydroxylation was reduced to 41%. Suppression of NDUFAF7 with 100 nm siRNA also reduced the relative level of hydroxylation of Arg-73 to a lesser extent (58%), but this reduction is the expected consequence of the extensive disruption of the assembly of complex I accompanying the suppression of expression of this assembly factor. The identities of the unmodified and hydroxylated forms of the AspN peptide (residues 66–79) containing Arg-73 were confirmed by fragmentation of the triply charged ions *m*/*z* 541.29 and 546.62, respectively (Fig. 3, *C* and *D*).

In cells where expression of NDUFAF5 had been suppressed, the extent of methylation of Arg-85 in NDUFS2 was unchanged, whereas the dimethylation was reduced progressively in cells where NDUFAF7 had been depleted (data not shown), as reported before (19).

##### Suppression of NDUFAF5 and Assembly and Function of Complex I

**FIGURE 4.**
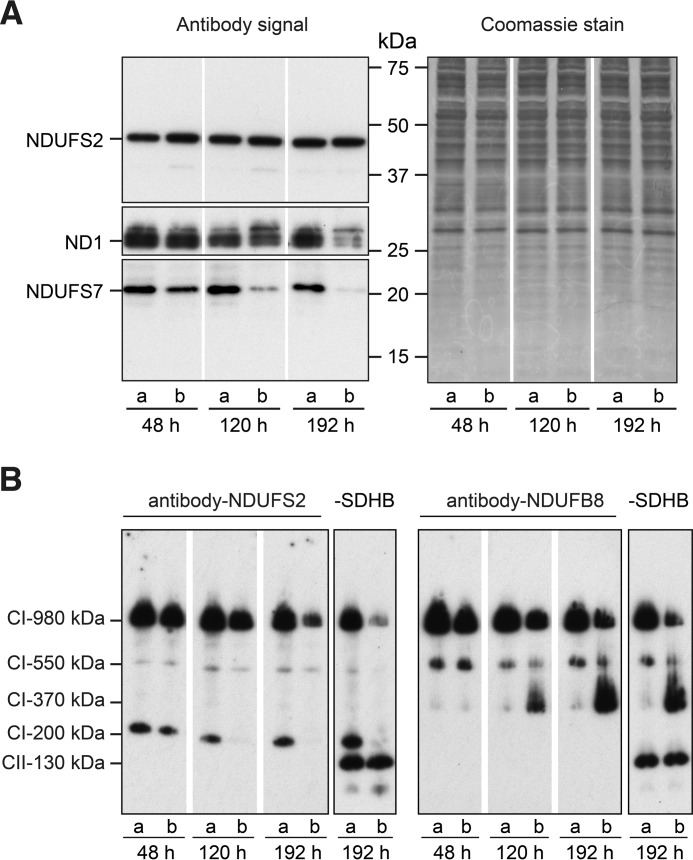
**Suppression of expression of NDUFAF5 and assembly of complex I.** Human 143B cells were transfected three times with either negative control siRNA or siRNA specific for NDUFAF5, denoted by *a* and *b*, respectively, and samples of mitoplasts and inner membranes were made 48 h after each transfection. *Parts A* and *B*, mitoplasts and inner mitochondrial membrane proteins, respectively, fractionated, in *A* by SDS-PAGE and, in *B*, by BN-PAGE. In *part A*, samples taken from three duplicate gels were Western-blotted with antibodies against subunits NDUFS2, NDUFS7, and ND1 of complex I, and one of them, employed as a loading control, was stained with Coomassie Blue dye, after Western blot analysis; in *part B*, membranes were probed with antibodies against the peripheral arm subunit NDUFS2, and membrane arm subunit NDUFB8, and probed a second time with an antibody against complex II subunit SDHB as a loading control, shown for the 192 h sample. *CI-980 kDa*, mature complex I; *CI-200 kDa*, −370 kDa, −550 kDa, sub-complexes of complex I; *CII-130 kDa*, complex II. The control cell results (denoted by the *letter a* in *A* and *B*) have been presented previously in another context (19).

When the expression of NDUFAF5 was suppressed, both NDUFS7 and ND1, representing the peripheral and membrane arms of complex I, were lost progressively (Fig. 4*A*), indicating that the participation of NDUFAF5 in the pathway of assembly of complex I affects both arms of the complex. Because endogenous NDUFS7 becomes degraded in these circumstances, the effect of suppression of expression of NDUFAF5 on the hydroxylation of Arg-73 in NDUFS7 was investigated in cells where NDUFS7 was overexpressed (Fig. 3). Although the level of subunit NDUFS2, a component of the peripheral arm, remained constant over the course of the experiment (Fig. 4*A*), the level of intact complex I was reduced progressively relative to control cells (Fig. 4*B*), indicating that some or all of the NDUFS2 being monitored had not been incorporated into the complex. The reduction in the level of complex I associated with the depletion of NDUFAF5 (Fig. 4*B*) was accompanied by the accumulation of a sub-complex with an apparent molecular mass of 370 kDa, containing subunit NDUFB8, a component of the membrane arm of complex I. At the same time, another sub-complex observed in control cells with an apparent molecular mass of 200 kDa, containing the peripheral arm subunit NDUFS2 was reduced.

**FIGURE 5.**
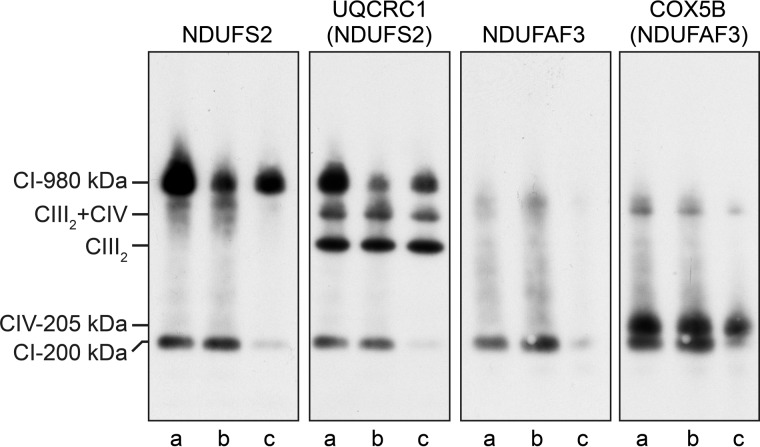
**Effect of transient suppression of expression of NDUFB3 and NDUFAF5 on the assembly of complex I.** Samples were prepared at 192 h from human 143B cells that had been transfected three times at 72-h intervals with (*a*), negative control siRNA or (*b* and *c*), siRNA specific for NDUFB3 and NDUFAF5, respectively. Inner mitochondrial membrane proteins were fractionated by BN-PAGE, Western-blotted and probed with antibodies against the peripheral arm subunit NDUFS2 and assembly factor NDUFAF3. Antibodies against complex III (UQCRC1) and complex IV (COX5B) were used as loading controls on NDUFS2 and NDUFAF3 blots, respectively. *CI*, complex I; *CIII_2_*, complex III dimer; *CIV*, complex IV; *CIII_2_+CIV*, assembly of complex III dimer and complex IV; *CI-200 kDa*, complex I sub-complex.

Subunit NDUFB3 contains methylated histidine residues, and therefore it was considered as a potential substrate for NDUFAF5. However, depletion of these two proteins impacted on different stages of the assembly of complex I. The suppression of NDUFAF5 affected the biogenesis of complex I at an early stage of assembly, whereas the depletion of NDUFB3 did not influence the formation of the early stage 200 kDa sub-complex identified with antibodies against NDUFS2 and assembly factor NDUFAF3 (Fig. 5). Additionally, NDUFB3 is a subunit of sub-complex Iβ, which represents the region of the membrane arm of complex I that lies distal from its peripheral arm (3, 14); this part of the complex is added at a later stage in the assembly process (18). Depletion of NDUFB3 had the effect of reducing the level of the intact complex (Fig. 5).

**FIGURE 6.**
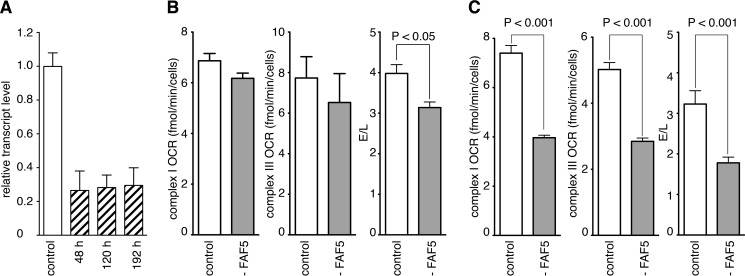
**Suppression of expression of NDUFAF5 and oxygen consumption.** Expression of NDUFAF5 in 143B cells was suppressed three times with siRNA at 72 h intervals. *Part A*, effect on levels of transcripts for control and NDUFAF5-depleted cells (white and hatched histograms, respectively) 48 h after each suppression. The values are normalized to endogenous β-actin, and error bars show the standard deviation. *Parts B* and *C*, oxygen consumption rates (*OCR*) of negative control and NDUFAF5 siRNA-treated cells (*white and shaded histograms*, respectively) 120 h (*B*) and 192 h (*C*) after the first transfection, respectively. OCR was normalized to cell number. Rates were measured after successive additions of 2-deoxyglucose, rotenone, duroquinol, and antimycin, or 2-deoxyglucose, oligomycin, carbonylcyanide *p*-(trifluoromethoxy)-phenylhydrazone (FCCP), and a combination of rotenone and antimycin A. The OCR for complex I represents the rate for 2-deoxyglucose-treated cells minus the rate for rotenone-treated cells, and those for complex III are the OCR values for duroquinol minus antimycin A. The E/L ratio (FCCP/oligomycin) is an index of the maximum oxygen consumption capacity of the electron transport system (*E*) relative to the magnitude of uncoupled respiration (*L*).

The effect of suppression of expression of NDUFAF5 on cellular energetics was examined by monitoring the OCR. Over the course of the experiment, transcripts for NDUFAF5 were reduced to about 30% of the control level (Fig. 6*A*). At 120 h, the suppression of NDUFAF5 was associated with a reduction of the E/L ratio (see Fig. 6*B*). At 192 h, the E/L ratio had been reduced further, and in these cells the OCR linked to complexes I and III was reduced (Fig. 6*C*). These effects are due to the disruption of the assembly of complex I and the potential secondary adverse effect of the depletion of NDUFAF5 on complex IV activity (21, 23). It has been noted before (21, 23) that the impact on complex IV may be cell/tissue specific, and although the reason for the effect is unknown it could be due to impairment of formation or stability of a supercomplex for example.

## Discussion

### 

#### 

##### NDUFAF5 as an Arginine Hydroxylase

**FIGURE 7.**
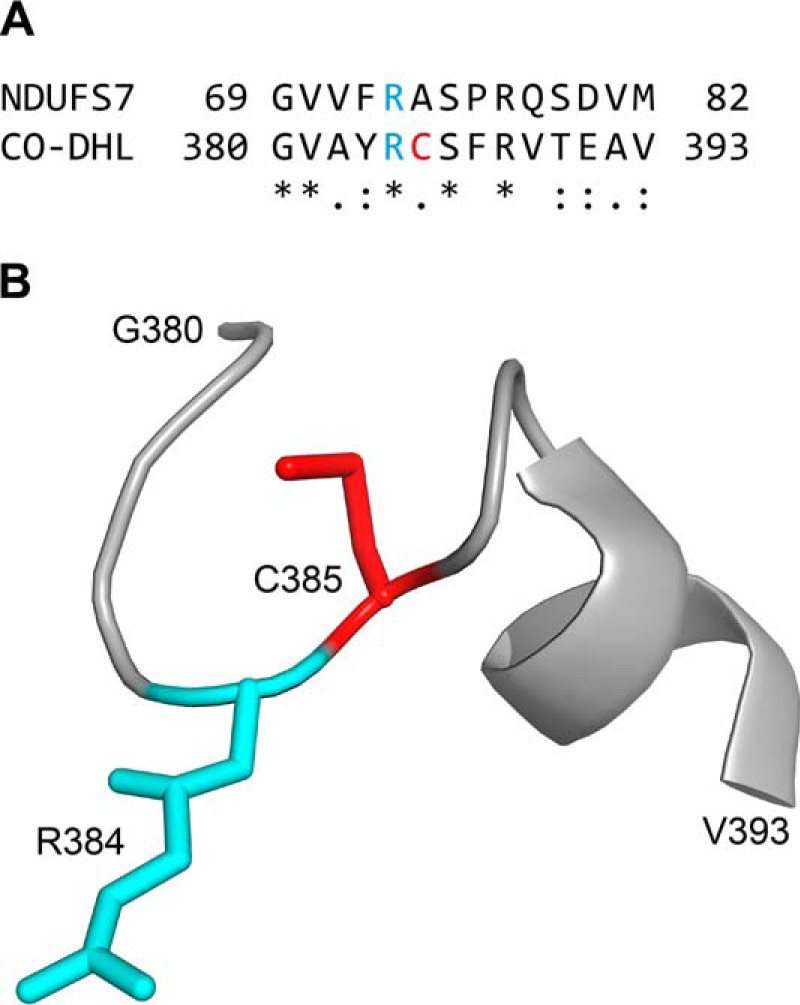
**Relationship between sites containing a hydroxylated-arginine residue in NDUFS7 and carbon monoxide dehydrogenase from *H. pseudoflava*.** *Part A*, alignment of sequences of residues 69–82 of human NDUFS7, and the active site loop of the carbon monoxide dehydrogenase large chain (CO-DHL; residues 380–393), with hydroxylated arginine residues in *blue*. In CO-DHL, the next residue (*red*) is the catalytically essential Cys-385. The symbols *, :, and . denote identical, strongly conserved, and weakly conserved residues, respectively; *part B*, the structure of the loop and the following α-helix containing residues 380–393 of CO-DHL. In the structures of bacterial and bovine complex I, the equivalent regions in the orthologs of NDUFS7 were not resolved.

The hydroxylation of arginine residues in proteins is a rare post-translational modification, and, other than NDUFS7, only three instances have been reported. First, a single 3-hydroxyarginine residue has been found at position-81 of the L16 component of the 50S subunit of the *Escherichia coli* ribosome (26). Second, numerous 4-hydroxyarginine residues are present in protein components of adhesive plaques of the mussel *Mytilus edulis* (27). Third, a single 4-hydroxyarginine residue has been identified in the large subunit of carbon monoxide dehydrogenase (CO-DHL) from *Hydrogenophaga pseudoflava* (28). From the point of view of the current investigation, the modification of CO-DHL is highly significant because the sequence around the site of hydroxylation in NDUFS7 is related to the sequence around the site of hydroxylation in CO-DHL (Fig. 7*A*). In the structure of CO-DHL, this sequence, which also includes the active site cysteine, forms a loop followed by an α-helix (28) (Fig. 7*B*). In bacterial and mitochondrial complexes I, the arginine residue is conserved, but the bacterial residue is not modified (25). There is no evidence that this arginine in prokaryotic complex I has a role in enzyme assembly or activity. NDUFS7 is the equivalent of bovine subunit PSST, fungal subunit NUKM and bacterial subunit NuoB/Nqo6. In none of the complex I structures was there unambiguous electron density for the loop (5, 9, 15), possibly because it is mobile. In bacterial complexes I, the corresponding arginine residue in subunit Nqo6 lies close to a tunnel, also involving subunits Nqo8 (ND1) and Nqo4 (NDUFS2), in the membrane and peripheral arms of the complex respectively, where the oxidized coenzyme Q is thought to bind and accept electrons from iron-sulfur cluster N2 (9). The restricted access to the residue via the tunnel in the mitochondrial enzyme makes it most unlikely that the hydroxylation could occur in this assembled state, in agreement with the proposal that the modification occurs during assembly of the complex, and before formation of the tunnel.

The finding that a member of the family of SAM-dependent 7β-strand methyltransferases is a hydroxylase has a precedent. While many protein and small molecule hydroxylases belong either to the 2-oxoglutarate dependent dioxygenases (29, 30), where the atoms of dioxygen are incorporated into two substrates, and not into water, or to the cytochrome P450 enzymes (31), which reduce one atom of dioxygen to a hydroxyl group and the other to water, another mono-oxygenase, RdmB, from *Streptomyces purpurascens* introduces a hydroxyl moiety into the 10-position of the anthracycline component of the anti-cancer drug aclacinomycin, or aclarubicin (32, 33). Like NDUFAF5, RdmB belongs to the family of SAM-dependent 7β-strand methyltransferases, and is structurally very similar to the methytransferase DnrK (the r.m.s.d. of superimposed main chain atoms is 1.14 Å) (34, 35). However, RdmB has no methyltransferase activity, and SAM acts as a cofactor in the process of hydroxylation (32, 33). Remarkably, the insertion of a single serine residue converted DnrK from a methyltransferase to a hydroxylase, causing the methyl group of SAM to point in a direction not suitable for methyl transfer (36). The positive charge of SAM is a crucial feature of the hydroxylation reaction as *S*-adenosylhomocysteine cannot substitute for SAM. Other requirements are dioxygen and a reductant such as glutathione.

**FIGURE 8.**
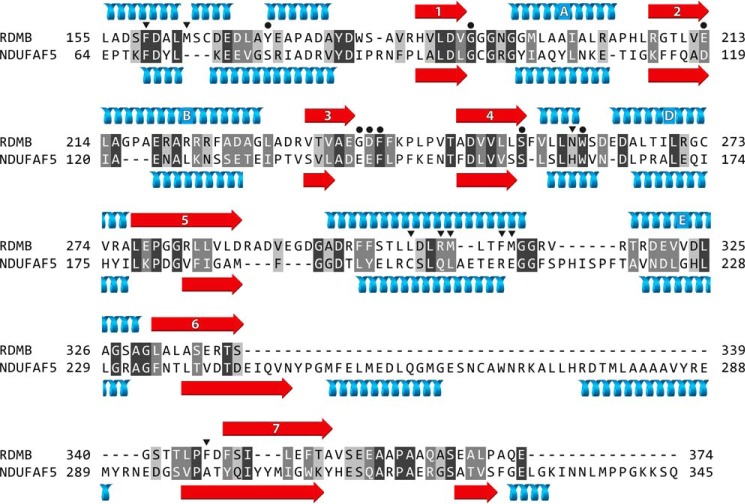
**Alignment of the C-terminal regions of the sequences of RdmB from *S. purpurascens* and human NDUFAF5.** The N-terminal regions (not shown) are unrelated. *White letters* on *dark gray* and *mid-gray* indicate identical and conserved residues, respectively, and *black letters* on *light gray* are weakly conserved residues. The secondary structural elements of the 7β-strand SAM binding domain of RdmB are shown *above* its sequence, and the predicted secondary structure of NDUFAF5 is shown *below*. Helices and β-strands are *blue* and *red*, respectively. The second helix of RdmB (residues 164–167) is a 3_10_-helix, and the others are α-helices. In RdmB, the 7β-strand methyl-transferase-fold consists of β-sheets 1–7 and intervening α-helices A–E. RdmB lacks helix C, found between β-strands 3 and 4 in some family members. The other α-helices lie outside the 7β-strand fold and contain residues involved in substrate binding. *Black dots* and *inverted black triangles*, respectively, denote amino acids involved in binding SAM, and in binding substrates and products.

The three-dimensional structure of RdmB has three domains. The N-terminal domain is involved in the formation of homo-dimers, the middle domain is mainly α-helical, and the C-terminal domain contains a Rossmann fold of five β-strands with associated α-helices on both sides, plus two additional β-strands to complete the characteristic 7β-strand fold (34). The substrate binds between the middle and C-terminal domains, and SAM to the C-terminal domain. The sequence of the region of RdmB containing the 7β-strand fold is related to the C-terminal region of NDUFAF5, and the predicted secondary structure of NDUFAF5 resembles the known secondary structure of RdmB over this region (Fig. 8). On this basis, it is likely that the mechanism of hydroxylation of Arg-73 in NDUFS7 by NDUFAF5 is similar to the mechanism of hydroxylation catalyzed by RdmB.

##### Role of NDUFAF5 in the Assembly of Mitochondrial Complex I

**FIGURE 9.**
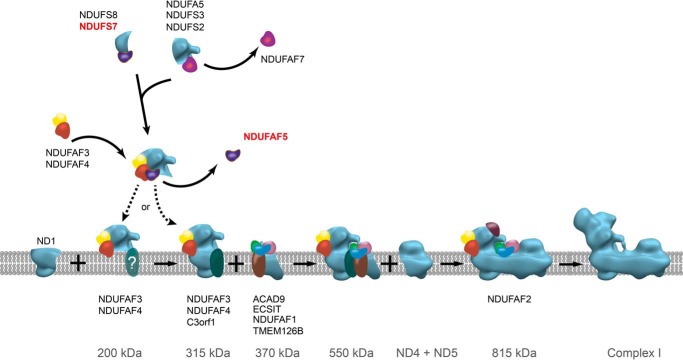
**Participation of NDUFAF5 in the pathway of assembly of human complex I.** Assembly factors associated stably with sub-complexes in the pathway are indicated. Hydroxylation of NDUFS7 by NDUFAF5 occurs at an early stage. The scheme is derived from an earlier version (19), and it is not known whether the 200-kDa sub-complex is associated with the membrane or not, as indicated. The 200-kDa sub-complex joins with membrane subunit ND1 and assembly factor C3orf1, plus subunits NDUFA3, NDUFA8, and NDUFA13 to form the 315-kDa membrane-bound sub-complex (13).

In human mitochondria, complex I is assembled from forty-four different proteins emanating from two genomes; the complex contains two copies of subunit NDUFAB1 and single copies of all other subunits (5). Thirty-seven of them are encoded and transcribed in the nucleus of the cell; they are translated in the cytoplasm and are imported into the organelle. The remaining subunits are encoded in mitochondrial DNA, and are the seven hydrophobic proteins that provide the core of the membrane arm of the complex. The assembly of human complex I proceeds via sub-complexes (16, 17), built up from specific subunits with the participation of exogenous proteins, known as assembly factors (13, 18) (Fig. 9). At least thirteen such assembly factors have been identified. Nine of them (NDUFAF1–4, ACAD9, C3orf1, ECSIT, FOXRED1, and TMEM126B) bind to specific sub-complexes in the assembly pathway (13, 37–42), but their exact molecular roles are unknown. Mutations in another group of assembly factors for complex I not found in association with assembly intermediates, lead to human disease associated with incompletely assembled complex I (21, 43–45). They include NUBPL and NDUFAF6. NUBPL probably participates in the incorporation of iron-sulfur clusters into the complex (46) and NDUFAF6 may help to stabilize the sub-complex of the peripheral arm that interacts with the membrane subunit ND1 (47). A twelfth assembly factor NDUFAF7 is a 7β-strand methyltransferase that dimethylates Arg-85 in the NDUFS2 subunit (19, 20). NDUFAF5 is a known thirteenth assembly factor, and the experiments described here show that its role in the process of assembly is to hydroxylate residue Arg-73 in subunit NDUFS7. They provide no support for an alternative proposal that during assembly of complex I, NDUFAF5 methylates histidines in the N-terminal region of subunit NDUFB3.

According to current models (see Fig. 9), a 315-kDa sub-complex is the earliest assembly intermediate involving components of both membrane and peripheral arms. It comprises at least eight nuclear encoded subunits (NDUFS2, NDUFS3, NDUFS7, NDUFS8, NDUFA3, NDUFA5, NDUFA8, and NDUFA13) plus one mitochondrial-DNA encoded subunit ND1 and three assembly factors (NDUFAF3, NDUFAF4, and C3orf1). The stages preceding the formation of this sub-complex involve the initiation of the assembly of the peripheral arm by NDUFS2, NDUFS3, and NDUFA5 joining with a NDUFS7-NDUFS8 sub-complex. A 200-kDa sub-complex containing at least NDUFS2 and assembly factor NDUFAF3 is dependent on NDUFAF5 (Figs. 4 and 5). Previously, the formation of a 200-kDa sub-complex was shown to be disrupted by depletion of assembly factor NDUFAF3 with similar results for assembly factors NDUFAF4, NDUFAF5, and NDUFAF6 (47). Residue Arg-85 in NDUFS2 is methylated by a transient interaction with NDUFAF7 at some point in the formation of the early peripheral arm sub-complex (19). In contrast, NDUFAF5 in the process of hydroxylating NDUFS7, is found associated with assembly factors NDUFAF3 and NDUFAF4 (Fig. 2*B*), as noted before (48). They are likely to participate in the formation of the early 200 kDa peripheral arm sub-complex formed only from NDUFS7, NDUFS2, NDUFS3, NDUFS8, and NDUFA5 (Fig. 9), as indicated by the preferential association of the four latter subunits with tagged-NDUFS7 (Fig. 2*B*). However, the precise point at which NDUFAF5 hydroxylates NDUFS7 in this process is not known. There is no indication in the data for a direct interaction between NDUFAF5 and MT-ND1, which participates in the formation of the 315 kDa intermediate (see Fig. 9). Therefore, NDUFAF5 must dissociate before its formation, as its presence would impede the association of the peripheral arm sub-complex with MT-ND1.

## Experimental Procedures

### 

#### 

##### Protein Analyses

Protein concentrations were determined by the bicinchoninic acid method. Samples containing proteins were analyzed by SDS-PAGE in 10–20% polyacrylamide gradient gels. Proteins were detected by staining with Coomassie Blue dye.

##### Cell Culture

Human 143B osteosarcoma cells (ATCC number CRL8303) were grown at 37 °C in Dulbecco's Modified Eagle's Medium (DMEM) containing 25 mm glucose and supplemented with fetal calf serum (10% *v*/*v*), penicillin (100 units/ml), and streptomycin (0.1 mg/ml) under an atmosphere of 5% carbon dioxide. The serum in the medium for parental human embryonic kidney cells HEK293T and HEK293T cells expressing Flag-Strep-tag II-fusion proteins was tetracycline-free, and the medium included blasticidin (15 μg/ml) and zeocin (100 μg/ml), or blasticidin (15 μg/ml) and hygromycin (100 μg/ml).

##### Protein Expression and Purification

The cDNA for human NDUFAF5 (Source Bioscience, Nottingham, UK) was amplified by polymerase chain reaction. It was cloned into the inducible expression vector pcDNA5/FRT/TO (Invitrogen) with sequences encoding C-terminal FLAG and StrepII tags. The cDNAs for human NDUFS7, METTL12, and NDUFB3 (Source Bioscience) were amplified and cloned in a similar way. The plasmid for NDUFAF5 was transfected into human 143B cells, and the subcellular location of the protein was examined by confocal microscopy (19). The transfection of HEK293T Flp-In T-REx cells with other plasmids, cell selection, and the preparation of mitoplasts and samples of Strep-tag II affinity-purified proteins, were performed as described previously (49).

##### Protein Quantitation with SILAC

Human HEK293T cells expressing either tagged-NDUFAF5 or NDUFS7, and with a potential mitochondrial protein methylase called METTL12 or with NDUFB3 as respective controls, were grown in “heavy” DMEM containing arginine and lysine isotopically labeled with ^15^N and ^13^C, and in “light” DMEM containing ^14^N and ^12^C arginine and lysine (Sigma) (50). These media were supplemented with penicillin (100 units/ml), streptomycin (0.1 mg/ml), proline (200 mg/liter), and dialyzed fetal calf serum (10% *v*/*v*). The cell population was doubled at least seven times. The expression of the tagged protein was induced for 24 h with doxycycline. Portions (*ca*. 10 mg protein) of the heavy and light cells were collected, mitoplasts were prepared, and proteins were purified from them by affinity chromatography. They were mixed and fractionated by SDS-PAGE, and proteins in slices taken from gel lanes were digested with trypsin (51). Tryptic peptides were analyzed by mass spectrometry (19, 49), and proteins were identified and quantitated with MaxQuant and Andromeda (52, 53). Bioinformatics and calculations of statistical significance were performed with Perseus (54). Each mass spectrometric ratio was based on duplicate SILAC experiments. Proteins that were associated in both experiments involving inducible and tagged versions of NDUFS7 and NDUFAF5 were considered to be significant binding partners.

##### Suppression of Expression

Transcripts for NDUFAF5 and NDUFB3 were suppressed transiently in 143B cells with 30 nm siRNA (Silencer Select siRNA, Life Technologies, Paisley, UK). A negative control siRNA (Allstars Negative Control siRNA; Qiagen, Manchester, UK) was used at the same concentration. Three successive transfections were performed at 72-h intervals. Transcript levels (normalized to endogenous β-actin) were determined by quantitative real-time PCR with a Taqman gene expression assay (Life Technologies) on cDNA prepared with a Cells-to-CT kit (Life Technologies). Samples of cells were taken 48 h after each transfection, and enriched either for mitoplasts or inner mitochondrial membrane proteins (19, 55).

Tagged NDUFS7 was overexpressed in HEK293T cells, and transcripts of NDUFAF5 or NDUFAF7 (used as a control) were suppressed transiently with 50 nm or 100 nm siRNA. Two successive transfections were performed at 72-h intervals, and 24 h after the second transfection, expression of tagged proteins was induced with doxycycline. Another 24 h later, proteins were affinity-purified, fractionated by SDS-PAGE, and detected with Coomassie Blue dye. Stained protein bands were digested with either trypsin or protease AspN, and subjected to mass spectrometric analysis.

##### Analysis of Hydroxylation of NDUFS7

Tryptic or AspN digests were analyzed in an LTQ OrbiTrap XL-mass spectrometer (Thermo Scientific). Peptides were fragmented by collision induced dissociation with nitrogen, or by electron transfer dissociation, as described previously (19, 49). The effect of the suppression of expression of NDUFAF5 on the hydroxylation of NDUFS7 was followed by analysis of the AspN peptide DRFGVVFRASPRQS (resides 66–79) where Arg-73 is hydroxylated. The monoisotopic *m*/*z* values for the unmodified and hydroxylated peptides are 811.4315 (M+2H)^2+^ and 541.2901 (M+3H)^3+^, and 819.4290 (M+2H)^2+^ and 546.6217 (M+3H)^3+^, respectively.

##### Effect of Depletion of NDUFAF5 on the Assembly of Complex I

Mitoplasts and inner mitochondrial membranes were prepared from 143B cells where NDUFAF5 had been depleted, and from control cells. The proteins were fractionated by SDS-PAGE and by blue native PAGE, and transferred by electrophoresis to polyvinylidene difluoride membranes as described previously (19). The levels of proteins, and the influence of suppression of expression of NDUFAF5 on the assembly of complex I, were assessed with antibodies against subunits NDUFS2 (Abcam ab96160), NDUFS7 (Abcam ab127051), NDUFB8 (Sigma HPA003886), and MT-ND1 (chicken anti-peptide antibody prepared against AETNRTPFDLAEGE; Agrisera, Vannas, Sweden), assembly factor NDUFAF3 (Sigma HPA035377), the SDHB subunit of complex II (Sigma HPA002868), UQCRC1 subunit of complex III (Sigma HPA002815) and COX5B subunit of complex IV (Sigma HPA034517).

##### Measurement of Respiration

The rate of consumption of oxygen (OCR) of 143B cells where NDUFAF5 had been depleted, and of control cells, was measured in an XF24 extracellular flux analyzer (Seahorse Biosciences, North Billerica) as described before (19). The assay medium was DMEM base formulation (Sigma) plus NaCl (1.85 g/liter), 2 mm glucose, 2 mm GlutaMax (Life Technologies), 1 mm pyruvate, phenol red (15 mg/liter), and 20 mm HEPES (pH 7.4). Cells in assay medium were equilibrated for 1 h at 37 °C in air. Then basal OCR was measured and either 2-deoxyglucose (20 mm), rotenone (600 nm), duroquinol (627 μm), and antimycin (600 nm), or 2-deoxyglucose (20 mm), oligomycin (100 nm), carbonylcyanide *p*-(trifluoromethoxy)phenylhydrazone (500 nm) and a combination of rotenone (600 nm) and antimycin A (600 nm), were added sequentially. Complex I-dependent OCR was determined by subtraction of rotenone-inhibited OCR values from values obtained after the addition of 2-deoxyglucose; complex III-dependent OCR was corrected by subtraction of antimycin A-inhibited OCR values from values obtained after the addition of duroquinol. *p* values for the OCR were calculated with a paired Student's *t* test.

## Author Contributions

J. E. W. designed research and supervised the project; V. F. R. and J. C. performed the experiments; S. D. and I. M. F. carried out mass spectrometry analyses; all authors participated in the analysis of data; J. E. W., V. F. R., and J. C. wrote the manuscript.

## Supplementary Material

Supplemental Data

## Footnotes

2 The abbreviations used are:

complex I: NADH:ubiquinone oxidoreductase
SILAC: stable isotope labelling by amino acids in cell culture
OCR: rate of consumption of oxygen
SAM: *S*-adenosylmethionine.
