# Supplementary material for: NDUFAF5 Hydroxylates NDUFS7 at an Early Stage in the Assembly of Human Complex I
Source: J Biol Chem. 2016 May 18;291(28):14851–60. doi: 10.1074/jbc.M116.734970 (PMC4938201; doi:10.1074/jbc.M116.734970)
Supplement: Supplemental Data [file supp_291_28_14851__index.html]

NDUFAF5 hydroxylates NDUFS7 at an early stage in the assembly of human complex I — NDUFAF5 Hydroxylates NDUFS7 at an Early Stage in the Assembly of Human Complex I — Assembly of Human Complex I — Supplemental Data 

# NDUFAF5 Hydroxylates NDUFS7 at an Early Stage in the Assembly of Human Complex I

## Supplemental Data

- Supplemental Tables 1 and 2 (.xlsx, 121 KB) - Proteins identified in SILAC experiments
